# Feasibility of breast conservation after neoadjuvant taxene based chemotherapy in locally advanced breast cancer: a Prospective Phase I trial

**DOI:** 10.1186/1750-1164-4-5

**Published:** 2010-08-31

**Authors:** Mohamed I El-Sayed, Doaa W Maximous, Mohamed A Aboziada, Mostafa E Abdel-Wanis, Nabiel NH Mikhail

**Affiliations:** 1 Department of Radiation Oncology, South Egypt Cancer Institute, Assiut University, Assiut, Egypt; 2 Department of Surgical oncology, South Egypt Cancer Institute, Assiut University, Assiut, Egypt; 3 Department of Biostatistics and Cancer Epidemiology, South Egypt Cancer Institute, Assiut University, Assiut, Egypt

## Abstract

**Background:**

Neoadjuvant chemotherapy is the standard care for locally advanced breast cancer. Our study aimed at evaluating the feasibility of breast conversation surgery (BCS) after neoadjuvant chemotherapy.

**Patients and methods:**

Forty five patients had stage IIB (except those with T2N1 disease) and stage IIIA were selected to 3 cycles taxane-based neoadjuvant chemotherapy. Patient who had tumours ≤5 cm underwent a tentative BCS while patients who had tumour size >5 cm underwent radical surgery. Negative margin is essential for BCS. Adjuvant chemotherapy and 3-D radiotherapy ± hormonal treatment were given to all patients.

**Results:**

Thirty four patients had BCS. Response to chemotherapy was the only statistically significant factor which influences the BCS. Incidence of local recurrence was 5.9% for patients who had BCS at a median follow up 24 months.

**Conclusion:**

Breast conservation is feasible in selected cases of locally advanced, non metastatic cancer breast. We recommend that patients who have tumour size ≤4 cm after chemotherapy are the best candidates for BCS.

## Introduction

Long-term survival is common among women with locally advanced breast cancer; therefore, quality of life issues become vitally important. For most women, loss of a breast is emotionally disturbing [1].

The patient's response to preoperative chemotherapy is a powerful prognostic factor in predicting disease-free and overall survival from locally advanced breast cancer [2,3]. By assessing the response to preoperative chemotherapy, it is possible to select out a better prognosis group of patients who will have improved long-term survival and a low rate of local recurrence. Therefore, patients who respond to preoperative chemotherapy are often the best candidates for breast conservation, allowing for less disfigurement with preservation of function [3,4].

Several studies have documented the feasibility and safety of breast conservation for locally advanced breast cancer after preoperative chemotherapy. Breast conservation is possible in 27% to 90% of patients after preoperative chemotherapy [4,5]. Local recurrence rates after breast conservation are low (5% - 10%) in patients who respond to preoperative chemotherapy [6,7].

Our primary outcome was the evaluation of the feasibility of breast conservation after neoadjuvant chemotherapy in patients with locally advanced, non metastatic breast cancer. Secondary outcomes were assessment of factors which might affect the feasibility of BCS and the status of surgical margins after breast conservation and determination of pattern of loco-regional recurrence, and common toxicity criteria among all patients after neoadjuvant chemotherapy.

## Patients and methods

This prospective phase I study was conducted at South Egypt Cancer Institute and Sohag cancer centre, Egypt, during the period from March, 2006 to September 2008. Each case was reviewed in the weekly interdisplinary tumour board before study inclusion, and all cases gave written informed consent. This study was approved by Assiut Faculty of Medicine Institutional Review Board that gives approval to both South Egypt Cancer Institute and Sohag Cancer Centre.

### (I) Eligibility criteria

A- At presentation: Female patients with biopsy proven locally advanced, non metastatic breast cancer {Stage IIB (limited to T3N0) and IIIA disease} and an Eastern Cooperative Oncology Group performance score of 0 to 1 with exclusion of multicentricity, diffuse microcalcification, central and retroareolar tumours, pregnancy and left ventricular ejection fraction (LVEF) of < 60%.

B- After neoadjuvant chemotherapy: Patients with tumours ≤5 cm in greatest dimension measured by clinical examination and both breast sonography and mammography.

Tumour: breast size ratio small enough for a good cosmetic result (subjective assessment) and no skin involvement.

C-After breast conservation: Patients with negative surgical margins.

### (II) Pre-treatment evaluation

Each patient was subjected to clinical examination, laboratory investigations (including complete blood picture, liver and renal function tests), echocardiography and breast sono-mammography. Axillary staging was done clinically and with ultrasound. True-cut needle biopsy was taken from breast tumor itself for histopathological diagnosis. Metastasis work up was done, such as chest x- ray, abdomino-pelvic ultrasonography and bone scan.

### (III) Neoadjuvant chemotherapy

Each patient was given 3 cycles of taxene based combination chemotherapy with 3 weeks interval, (Paclitaxel 135 mg/m^2^, Adriamycin 50 mg/m^2 ^and Cyclophosphamide 500 mg/m^2^). Three weeks after the third cycle, each patient was evaluated by clinical examination and breast sono-mammography to assess the response to neoadjuvant chemotherapy. Axillary disease was evaluated by physical examination and by ultrasound. Chest x- ray and abdomino-pelvic ultrasound were done to exclude patients with distant metastasis.

### (IV) Treatment categorization

Patients with tumours ≤5 cm in greatest dimension underwent conservative breast surgery (wide local excision with 2 cm safety margin and axillary dissection). Patients with breast tumours > 5 cm in greatest dimension or those with persistent positive surgical margins were excluded from the study and were subjected to modified radical mastectomy (MRM).

### (V) Postoperative adjuvant therapy

Each patient in both groups was given additional 3 cycles of taxene based combination chemotherapy followed by radiotherapy (RT) of 50 Gy with 2 Gy daily fractions to breast and chest wall using 3-D planning by 2 parallel opposed tangential fields using 6 MV photon beams. Supra-clavicular irradiation (50 Gy/25 fractions/5 weeks) was given only to patients with positive axillary lymph nodes. A boost dose of 16 Gy in 8 fractions to tumour site using 12 Mev electrons was given to patients who underwent conservative surgery.

Adjuvant hormonal therapy (tamoxifen, 20 mg/day, in 2 divided doses) was given only to patients with positive hormonal receptors.

Patients had multiple CT cuts at 1 cm interval throughout the treatment volume. At each CT slice, the target volume, heart, ipsilateral and contralateral lungs were defined. The dose volume histogram for the target volume and critical organs were obtained for treatment plan evaluation. The treatment plan was acceptable if ≤10% of the heart volume and ≤25% of the ipsilateral lung volume received 25 Gy [8].

### (VI) Follow up

All patients in this study (in both groups) were followed up monthly by clinical examination and every 3 months by sono-mammography to diseased and healthy breasts, as well as by chest x- ray and abdomino-pelvic ultrasound. Treatment related complications were measured using WHO common toxicity criteria [9].

### Statistical methods

Data was analyzed using SPSS version 17. Data was presented as frequencies and percentages (categorical data) and as mean ± SD (quantitative data). Statistical analysis was done using Chi square test (categorical data) and Wilcoxon signed-rank test (quantitative data). P < 0.05 was considered significant.

## Results

The majority of our patients (27 out of 45 patients; 60%) were < 50 years of age , 22 of them were eligible for BCS. The patients' age ranged from 35 to 69 years with the median age of 48 years. Forty one patients (91%) had large tumour size (> 5 cm); none of them were fixed to pectoralis major muscle nor to chest wall; 10 patients of them had no palpable ipsilateral axillary nodes (T3N0), 25 patients had mobile ipsilateral axillary nodes (T3N1) and only 6 patients had fixed nodes; table 1.

**Table 1:** Patients' characteristics

	*Baseline characteristics of all patients (n = 45)*		Patients who were eligible for BCS (n = 36)	
	**N**	**%**	**N**	**%**
**Age at diagnosis**				
< 50	27	60	22	61.1
≥50	18	40	14	38.9
**Tumour size**				
≤5 cm	4	8.9	4	11.1
> 5 cm	41	91.1	32	88.9
**Tumour site**				
Upper outer quadrant	32	71.1	26	72.2
Lower outer quadrant	9	20	7	19.4
Upper inner quadrant	4	8.9	3	8.3
**Laterality**				
Right sided	24	53.3	19	52.8
Left sided	21	46.7	17	47.2
**Clinical staging**				
T3N0	10	22.2	8	22.2
T3N1	25	55.6	20	55.6
T3N2	6	13.3	4	11.1
T2N2	4	8.9	4	11.1
**Histologic grade**				
Grade II	30	66.7	25	69.4
Grade III	15	33.3	11	30.6
**Hormonal status (HR)**				
HR positive	37	82.2	30	83.3
HR negative	8	17.8	6	16.7

**Total**	45	100	36	100

**Figure 1:**
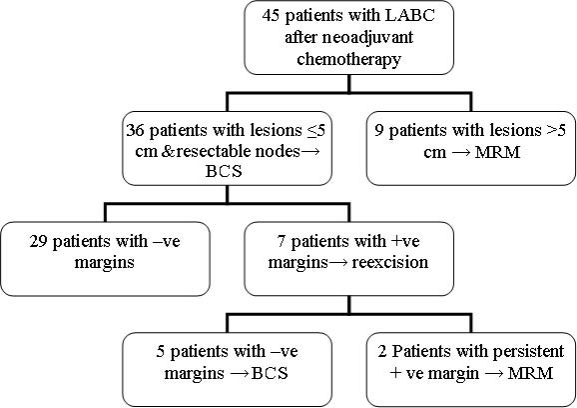
**The profile of the study**.

The profile of this study (figure 1) showed that, after neoadjuvant chemotherapy, 36 out of 45 patients had partial response (80%) with lesions ≤5 cm and underwent BCS, 29 patients of them (80.6%) showed negative (negative) surgical margins and the other 7 patients had positive margins. With re-resection, five out of the 7 patients had negative margins and the remaining 2 patients had persistent positive surgical margins. These 2 patients as well as the 9 patients with stable disease after neoadjuvant chemotherapy underwent MRM. Thus only 34 out 45 patients (75.6%) underwent BCS with negative surgical margins. Out of 10 patients (T3N0) with clinically impalpable and sonographically non detectable axillary nodal disease, 5 patients (50%) showed pathologically positive nodal disease after neoadjuvant chemotherapy and axillary dissection. Fifteen out of 35 patients (42.9%) with clinically documented axillary nodal disease (25 patients with T3N1, 6 with T3N2 and 4 with T2N2) showed complete clinical nodal response. All these 15 patients had stage T3N1 disease. Patients with clinically fixed axillary nodes (10 patients; 6 of T3N2 and 4 of T2N2 disease) showed only partial response.

The relation of clinical staging with grade and hormonal (HR) status had been studied after neoadjuvant chemotherapy; table 2. Staging, grade, and HR status were not statistically significant (> 0.05) on the feasibility of BCS. On comparison of pre-chemotherapy and post chemotherapy tumour sizes, there was statistically significant decrease of tumour size after neoadjuvant chemotherapy; table 3.

**Table 2:** Relation of Clinical staging with grade and hormonal (HR) status showing feasibility of BCS after neoadjuvant chemotherapy

Clinical Stage	N	Grade		HR	
		
		G II	G III	HR +	HR -
**T3N0**	10 (8)	8 (6)	2 (2)	9 (8)	1 (0)
**T3N1**	25 (20)	16 (12)	9 (8)	22 (17)	3 (3)
**T3N2**	6 (4)	4 (2)	2 (2)	3 (1)	3 (3)
**T2N2**	4 (4)	2 (2)	2 (2)	3 (3)	1 (1)

**Total**	45 (36)	30 (22)	15 (14)	37 (29)	8 (7)

**N.B.: **Numbers between brackets refer to numbers of patients whose post chemotherapy tumour size ≤5 cm and were candidates for BCS in different stage groups according to grade and HR status.

**Table 3:** Comparison of pre-chemotherapy tumour size and post chemotherapy tumour size

*Pre-chemotherapy tumour size*	*Post-chemotherapy tumour size*	P value*
**6.4 ± 1.4 cm**	3.9 ± 1.3 cm	< 0.001

* Wilcoxon signed-rank test

Out of 36 patients who did BCS 7 patients had positive margins (19.4%) and 29 patients had negative margins as distributed in table 4. The feasibility of BCS with negative margins was statistically insignificant as regard of the stage, grade, and HR status. All patients with post chemotherapy tumour size <4 cm underwent BCS with negative surgical margins whereas only 46.2% of those (6 out of 13 patients) with post chemotherapy tumour size between 4 and 5 cm showed negative margins. Therefore, post chemotherapy tumour size was the only statistically significant factor (P < 0.001) affecting the feasibility of BCS with negative margins.

**Table 4:** Relation between clinical staging with grade, hormonal (HR) status and post chemotherapy tumour size showing status of surgical margin in patient who did BCS

Clinical Stage	N	Grade		HR status		Post-chemotherapy tumour size	
		
		G II	G III	HR +	HR -	< 4 cm	4-5 cm
**T3N0**	8 (6)	6 (5)	2 (1)	8 (6)	0 (0)	5 (5)	3 (1)
**T3N1**	20 (17)	12 (10)	8 (7)	17 (15)	3 (2)	12 (12)	8 (5)
**T3N2**	4 (2)	2 (0)	2 (2)	1 (0)	3 (2)	2 (2)	2 (0)
**T2N2**	4 (4)	2 (2)	2 (2)	3 (3)	1 (1)	4 (4)	0 (0)

**Total**	36 (29)	22 (17)	14 (12)	29 (23)	7 (6)	23 (23)	13 (6)

**N.B.: **Numbers between brackets refer to numbers of patients who did BCS with free surgical margins in different stage groups according to grade, HR status and post chemotherapy tumour size.

The median follow up period was 24 months (range; 21-30 months). There were 2 patients who underwent BCS developed local recurrence (LR) (5.9%) at the tumour bed. One of the two patients had also simultaneous liver metastases (Distant metastasis rate of 2.9%) at 18 months. This patient was treated by salvage chemotherapy (Navelbine/5-flurouracil regimen). The other patient had only isolated LR at 20 months. This patient was treated by salvage MRM. The patient who had isolated LR was T3N1M0 disease at presentation with grade III and HR negative breast cancer. The other patients who had total disease relapse (LR and DM) were T3N2M0 disease at presentation with grade III and HR negative tumour. There was only one patient who underwent MRM (one out of 11 patients; 9.1%) developed LR at operative scar and this recurrent nodule was surgically excised. Distant metastasis did not occur in mastectomized patients.

The most common toxicity criteria following neoadjuvant chemotherapy among our patients was fatigue (40 patients; 88.9%) followed by grade 2 alopecia (38 patients; 84.4%). Athralgia and myalgia were developed in 8 patients (17.8%) whereas febrile neutropenia occurred in only 3 patients (6.7%); table 5. All patients completed the treatment protocol without interruption of treatment. There were no RT pneumonitis, and no severe cardiac toxicity occurred among the patients accrued to this study. The only radiation induced skin toxicity encountered was dry desquamation which did not necessitate interruption of radiation therapy.

**Table 5:** Common toxicity criteria among all patients after neoadjuvant chemotherapy

*Variable*	Chemotherapy toxicity	
	
	N	%
Fatigue	40	88.9
Alopecia	38	84.4
Arthralgia and myalgia	8	17.8
Diarrhoea	5	11.1
Mouth sores	4	8.9

**Febrile neutropenia**	3	6.7

## Discussion

This study emphasizes two important points: first, the high feasibility of BCS after neoadjuvant chemotherapy, and second, the good tolerability of patients to treatment with low incidence of grade 3 and grade 4 toxicity.

In the present study, after neoadjuvant chemotherapy, 36 out of 45 patients showed partial response with post chemotherapy tumour size ≤5 cm. This figure of response is within the range of the reported studies where Newman et al. [10] found that 75% of patients were feasible to BCS. The overall objective response of the primary tumour in patients with locally advanced breast cancer ranged from 71% to 87% [11,12]. On the other hand, our results are much higher than that reported by Yadav et al. [13] who found that only 23% of patients with locally advanced breast cancer were candidates for BCS after neoadjuvant chemotherapy. This difference could be explained on the ground that the reported study used anthracycline based chemotherapy (FAC regimen) while in the current study we used taxene based chemotherapy (TAC regimen).

The vast majority of our patients (41 patients, 91%) had large tumour size (> 5 cm), 10 patients of them (22.2%) had T3N0 (stage IIB), 25 patients (55.6%) had T3N1 (stage IIIA) and only 6 patients (13.3%) had fixed nodes (stage IIIA). Our figures are different than those reported by Formenti et al. [14] where 36% patients had stage IIB (T3N0) tumours, 30% had stage IIIA, and 34% had stage IIIB. This may be due to exclusion of stage IIIB cases in our study.

The mean prechemotherapy tumour size was 6.4 ± 1.4 cm in the present study which was similar to that reported by Chen et al. [15] where the median tumour size before chemotherapy was 6.0 cm. Our figure is also comparable with that reported by Viswambharan et al. [12] where the size of the primary tumour ranged from 5-9 cm (mean = 7.1 cm). The mean post-chemotherapy tumour size in our study, was 3.9 ± 1.3 cm which was comparable to figures reported by Chen et al. [15] (2.2 to 3.2 cm) and Viswambharan et al. [12] (3.8 cm).

In the current study, we found that stage, grade, and HR were not statistically significant on the response to chemotherapy. However, the reported series showed that nuclear grade[16] and HR negative status[17,18] were significantly related to tumour response to preoperative chemotherapy. This difference may be explained by the larger number of patients in the reported series (287 patients in Abu-Farsakh et al. [16], 60 patients in Sarid et al. [17], and 399 patients in Collini et al. [18]). All patients with post chemotherapy tumour size <4 cm underwent BCS with negative surgical margins whereas only 46.2% of those (6 out of 13 patients) with post chemotherapy tumour size between 4 and 5 cm were feasible for BCS with negative margins. This is in agreement with Neuman et al. [10] who stated that post-chemotherapy tumour size less than 4 cm is a favourable criterion regarding the feasibility of BCS.

The rate of positive margins, in our study, was 19.4% (7 out of 36 patients) which was higher than that observed by Mittra et al. [19] where only 2.4% of patients with BCS showed positive margins. This difference may be explained by the larger number of patients in their study (726 patients) than in our study (36 patients).

All patients with post chemotherapy tumour size <4 cm underwent BCS with negative surgical margins whereas only 46.2% of those (6 out of 13 patients) with post chemotherapy tumour size between 4 and 5 cm showed negative margins. After re-resection most of patients with positive margins (5 out of 7 patients; 71.4%) achieved negative margins, but with compromised cosmetic appearance. This can be explained on the ground that, there was a significant association between pre-chemotherapy tumour size and histopathological margin status. There was also, a significant association between post-chemotherapy tumour size and histopathological margin status[12]. In patients with post-chemotherapy tumour size >4 cm, 10/13 (77%) were margin positive and 3/13 (23%) were margin negative whereas in patients with post-chemotherapy size >3 cm, 13/24 (54%) were margin positive and 11/24 (46%) were margin negative. In patients with post-chemotherapy size <3 cm, 1/6 (17%) were margin positive and 5/6 (83%) were margin negative. Therefore a smaller post-chemotherapy size is found to give lesser margin positivity and better cosmetic appearance. Therefore the recommendation for breast conservation surgery was for tumours up to 4 cm[20].

In this study, the median follow up time from the date of registration in outpatient clinic was 24 months (range 21-30 months). The relapse rate among patients who underwent conservative surgery was 5.9% (2 out of 34 patients). This figure is identical to that reported by Mittra et al. [19] who found a 5.9% relapse rate among patients in the conservative surgery group. Another study from the M. D. Anderson Cancer Centre evaluated outcome after induction chemotherapy and BCT for 93 patients with locally advanced or large primary breast cancers and found a local recurrence rate of <10%. This was comparable to the local recurrence rate seen in patients with early-stage breast cancer treated with a breast-conserving approach[21].

It is reassuring to note that the tolerance to preoperative chemotherapy was very good. Adverse effects were mostly of grade 1 or 2 severities. Grade 4 leucopenia was observed in only 3 cases. The most common toxicity criteria following neoadjuvant chemotherapy among our patients was fatigue (88.9%). This is matched with Sarid et al. [17] who found that adverse effects were mostly of grade 1 or 2 and the most common side effect was fatigue. Alopecia was observed in 84.4% of cases. This is comparable with Formenti et al. [14] who observed a 92.7% rate of alopecia in their study (38 of 41 patients). Athralgia and myalgia developed in 17.8%, diarrhoea in 11.1% and mouth sore in 8.9%. Febrile neutropenia (who were admitted for treatment) occurred in only 6.7% of patients. Formenti et al. [14] found similar results where arthralgia was observed in 17%, stomatitis in 12%, and febrile neutropenia in 9.7% of patients. On the other hand, Chen et al. [15] found a lower incidence of febrile neutropenia (2%) which may be due to their use the regimen of weekly taxol which is more tolerable with fewer side effects.

It is interesting that no RT pneumonitis occurred among the patients accrued to this study. There was also no severe cardiac toxicity. The finding is consistent with the University of Washington's experience and is in contrast with that reported by Yu et al. [22] who observed RT pneumonitis in 19% of 21 breast cancer patients treated by concurrent paclitaxel and RT and 20% of 16 patients who received RT after paclitaxel. Both groups of patients, however, were treated postoperatively and after doxorubicin-based chemotherapy. It is possible that the sequencing of anthracycline-based chemotherapy and taxanes affects the pulmonary morbidity of subsequent RT therapy[14]. The absence of radiation related pulmonary toxicity in our series, may also be attributed to the use of 3-D radiation therapy planning for our patients.

## Conclusions and recommendations

Breast conservation is feasible in selected cases of locally advanced, non-metastatic cancer breast, after neoadjuvant chemotherapy. Response to chemotherapy is the most important factor to select patients for BCS. We recommend that patients who have tumour size ≤4 cm after chemotherapy are the best candidates for BCS.

## Competing interests

The authors declare that they have no competing interests.

## Authors' contributions

ME-S, WM, AA, EA-W and NNHM participated in the patient diagnosis, management and the manuscript writing. All authors read and approved the final manuscript.
